# Assessing the Accuracy and Precision of Artificial Intelligence for Diabetes Mellitus and Hypertension Management

**DOI:** 10.3390/jcm15124419

**Published:** 2026-06-07

**Authors:** Abdullah Al Hamid, Reema Alshahrani, Maryam Alghareeb, Hadeel Hawsawi, Renad Alghareeb, Monther Abdolmohsin Alsultan

**Affiliations:** Department of Pharmacy Practice, College of Clinical Pharmacy, King Faisal University, Al Ahsa 36362, Saudi Arabia; reemma2021@gmail.com (R.A.); maymounah789@gmail.com (H.H.); maryam.alghareeb00@gmail.com (M.A.); renad.alghareeb0@gmail.com (R.A.)

**Keywords:** artificial intelligence (AI) tools, diabetes mellitus, hypertension, guidelines

## Abstract

**Background/Objectives**: Diabetes mellitus and hypertension are major chronic conditions that markedly affect patients’ health and quality of life worldwide. With the rapid development of technology, there has been a growing interest in exploring the potential role of artificial intelligence (AI) in the management of such diseases. This study aims to assess the accuracy and reliability of artificial intelligence tools in providing information for diabetes mellitus and hypertension management. **Methods**: This study assessed the accuracy and reliability of the information provided by major AI tools such as ChatGPT, Gemini, POE, Claude, Consensus, and Perplexity. Twenty questions that are essential for the management of diabetes mellitus and hypertension were constructed based on the chapters of the respective guidelines and were fed to the AI tools. The outcomes were compared with evidence-based treatment guidelines, such as those from the American Diabetes Association (ADA), the American Heart Association (AHA), the European Society of Cardiology (ESC), and the National Institute for Health and Care Excellence (NICE). Answers were classified into “accurate “, “inaccurate”, and “accurate with missing information”. Three rounds of six-week intervals were conducted to assess accuracy and reliability. In addition, they were conducted to evaluate data updates by comparing answers across the rounds. **Results**: In round one of the evaluations, ChatGPT and Poe showed the highest accuracy, both at 65% (95% CI: 41.0–83.7), followed by Claude at 60% (95% CI: 41.0–83.7). ChatGPT had the lowest inaccuracy rate at 5% (95% CI: 1.75–33.1), while Claude demonstrated the smallest percentage of responses with missing information at only 6%. (95% CI: 12.8–54.3). In round 2, Claude markedly outperformed all other tools, achieving an accuracy rate of 95% (95% CI: 73.0–99.7) and no responses with missing information (0%). In round 3, ChatGPT came second with 70% (95% CI: 45.70–87.2) accuracy and maintained the lowest inaccuracy rate of 5% (95% CI: 0.26–26.9). Consensus had the largest inaccuracy rate at 40% (95% CI: 20.0–63.6) and the lowest accuracy rate at 40% (95% CI: 20.0–63.6). Overall, statistically significant pairwise comparisons showed that Cloud in the second round has the highest accuracy compared to Poe (*p* = 0.0154), Gemini (*p* = 0.0421), Consensus (*p* = 0.0035), and Perplexity (*p* = 0.0302). In the assessment of performance shift from round 1 to round 2, Claude achieved the greatest improvement in accuracy at 40%. In the assessment of performance shift from round 2 to round 3, Poe improved the most with an accuracy increase of 25%, while ChatGPT followed with 20%. When evaluating the unprompted and guideline-prompted questions for all AI tools using McNemar’s test, it did not reveal a statistically significant distinction in the proportion of accurate responses (*p* > 0.05). **Conclusions**: Throughout the three rounds, ChatGPT maintained the best performance, with the fewest missing data. Claude and Poe followed, showing high accuracy with relatively low inaccuracy rates. On the other hand, Perplexity and Gemini performed moderately, while Consensus had the lowest accuracy.

## 1. Introduction

Diabetes mellitus and hypertension are major chronic conditions that markedly affect patients’ health and quality of life worldwide. According to the World Health Organization (WHO), in 2022 [1,2], 14% of adults aged 18 years and older were diagnosed with diabetes, an increase from 7% in 1990. Recent studies show that diabetes and kidney disease due to diabetes caused over 2 million deaths. Furthermore, hypertension is a leading cause of premature death globally; it is one of the major causes of cardiovascular disease and stroke. A report from the WHO shows that an estimated 1.28 billion adults aged 30 to 79 years are affected by hypertension worldwide.

Recently, artificial intelligence (AI) tools have notably developed and transformed how we analyze data, make decisions, and solve complex problems. AI tools are defined as software applications or platforms that use artificial intelligence algorithms to perform tasks that typically require human intelligence, including learning and analyzing large amounts of data [3,4].

A recent study reveals that AI tools can assist with clinical practice by supporting healthcare professionals in diagnosis, outcome prediction, treatment planning, and population health management. Moreover, AI possesses the capacity to enhance the quality, efficiency, and efficacy of healthcare services by giving timely and personalized information to facilitate decision-making [5]. Moreover, AI may assist clinical practice by advancing the overarching healthcare objectives of improving population health, enhancing patient and clinician experiences, and decreasing healthcare expenditures [6]. Nevertheless, the potential advantages underscore the necessity of rigorously evaluating the quality and comprehensiveness of AI-generated solutions prior to their broader application in clinical or educational healthcare environments.

The most widely used AI tools, such as ChatGPT, Gemini, Poe, Perplexity, Consensus, and Claude, are designed to process language and deliver information, recommendations, or support based on user-submitted questions. The underlying technology of these AI tools is natural language processing (NPL) [7]. NPL is fundamentally a developing algorithm and model that enables AI tools to comprehend human speech and text, which allows them to perform tasks such as chatbot interaction and language translation and provide a natural human response. Another technology that is essential to AI is machine learning [8]. It is a branch of artificial intelligence, enabling computers to learn from data and enhance their performance over time. It involves three main types: supervised, unsupervised, and reinforcement learning. Hence, the above-mentioned AI tools are considered Large Language Models (LLMs) and use a multimethod approach, including supervised learning and reinforcement learning [9]. Supervised learning involves training models on labeled data, where the algorithm learns to predict outcomes based on input–output pairs, usually used in tasks like image recognition. Unsupervised learning handles unlabeled data; it identifies hidden patterns or groupings. Reinforcement learning relies on trial and error, where the model interacts with an environment and learns by receiving rewards for correct actions and punishment for incorrect ones; this is often applied in robotics and gaming [10].

It is evident that patients and medical professionals depend on different online platforms to obtain medical information. Therefore, research on these platforms has a growing interest due to the development of medical information sources into these digital spaces [11]. The distribution of health information through social media platforms such as YouTube, Instagram, and TikTok has been the topic of research over the past decade. However, this research has revealed that the medical information found in these platforms is often unreliable and has a high rate of error [11]. In recent years, AI chatbots have been progressively used in medicine, especially ChatGPT, which draws attention with more than 170 million users globally [12]. Alongside ChatGPT, there are many other AI chatbots, such as Perplexity, Claud, Consensus, Poe, and Gemini. With the emergence of AI and its adoption into the healthcare field, the potential application of patient health data has generated significant interest. Although chatbots can deliver authoritative answers to complex medical questions, despite these findings appearing reliable, they are, in many aspects, inaccurate and raise concerns requiring caution. Moreover, the reliability and accuracy of these AI tools are not thoroughly assessed for open-ended medical questions [13].

In this paper, our aim was to design an exploratory evaluation of AI-generated responses to clinical questions related to diabetes and hypertension management. This study qualitatively evaluates the performance of AI tools such as ChatGPT, Gemini, Poe, Perplexity, Consensus, and Claude to assess the accuracy and reliability in responding to clinical questions about diabetes mellitus and hypertension management obtained from clinical guidelines chapters.

## 2. Materials and Methods

Twenty open questions were formulated based on chapters from multiple guidelines in the interest of comprehensiveness, including such guidelines as the American Diabetes Association (ADA) [14], (AHA) [15], the European Society of Cardiology (ESC) [16], and the National Institute for Health and Care Excellence (NICE) [17], to address the management of diabetes mellitus and hypertension. The questions were designed to thoroughly evaluate clinically pertinent elements of diabetes mellitus and hypertension therapy, involving diagnosis, pharmacological treatment, monitoring, dosage adjustment, comorbidity management, and treatment optimization. The study team formulated and evaluated the questions to guarantee clarity, clinical significance, and alignment with guideline-based recommendations. Then, the questions were internally evaluated for suitability and clarity before their application in the study.

To guarantee methodological consistency, the same prompts with identical wording were utilized in all three evaluation rounds. Each clinical question was presented verbatim in each round, without any changes to the structure, language, or context. Any variance in the AI-generated responses would therefore be due to differences in the model’s performance, not differences in the input.

The evaluators categorized each response by comparing it with the primary recommendations outlined in the diabetes and hypertension guidelines, which were used to formulate the clinical questions (ADA, AHA, ESC, and NICE). This comparison process was intended to assess if the AI output adhered to the established clinical standard. Then, the responses were classified as follows:

- “Accurate”: The response completely adhered to the guideline recommendations, featured no clinically incorrect statement, and addressed all critical components pertinent to the question.
- “Accurate with missing information”: The response provided correct guidance consistent with the referenced guidelines, but it lacked one or more essential elements necessary for a complete or comprehensive answer.
- “Inaccurate”: The response included recommendations that were contradictory with guideline recommendations, clinically unsuitable suggestions, or significant errors that made the overall guidance unreliable.

It is worth mentioning that during the preparation of this manuscript, the authors used ChatGPT-5 (OpenAI, San Francisco, CA, USA) for language proof reading and editing purposes. The authors reviewed and edited the output and take full responsibility for the content of this publication. 

### 2.1. Example 1

The American Diabetes Association (ADA) statement: section “Management of preexisting type 1 diabetes and type 2 diabetes in pregnancy”; insulin should be used to manage type 1 diabetes in pregnancy. A suitable insulin is the preferred agent for the management of type 2 diabetes in pregnancy.

Question version of the statement: What are the medications recommended for DM in pregnant persons?

### 2.2. Example 2

The European Society of Cardiology (ESC) statement: section “8.3.2. Drug classes with evidence on clinical outcomes in the target population: the major drug classes with robust evidence for BP-mediated reduction in CVD events are ACE inhibitors, ARBs, dihydropyridine CCBs, diuretics (thiazides and thiazide-like diuretics such as hydrochlorothiazide, chlorthalidone, and indapamide), and beta-blockers”.

Question version of the statement: What are the most common contraindications for HTN medications?

The questions posed to open AI tools such as ChatGPT, Gemini, Poe, Perplexity, Consensus, and Claude (all were open, free versions) were conducted in three rounds of six-week intervals. The first round started on 31 August 2024, followed by a second round on 5 October 2024, and concluded with the final round on 14 November 2024. Each round was conducted to assess accuracy and reliability. In addition, they were meant to evaluate the level of data updating by comparing the data across the rounds. Furthermore, we extended the capability of the AI tools by adding “according to…” and providing specific references, including the American Diabetes Association(ADA) and the American Heart Association (AHA), to assess their effectiveness in enhancing the accuracy of AI-generated answers by explicitly anchoring the responses to reputable evidence-based sources, with a focus on diabetes and hypertension management guidelines.

The responses were exchanged between the two clinical pharmacists after the categorization to ensure the validity of the categorization process for each answer. Any disagreements were resolved through discussion between the two clinical pharmacists. The responses were then classified into their appropriate category and organized in a table.

For assessing and evaluating the ability of the AI tools to update their responses throughout three rounds, the established criteria included:

- “Improved accuracy”: when a previously inaccurate or accurate response with missing information became accurate. Also, if inaccurate responses became accurate with missing information.
- “Consistency”: where responses remained accurate across the three rounds.
- “No improvement”: where no changes in response were noticed over rounds.
- “Decline in accuracy”: when accurate responses decreased to accurate with missing information or inaccurate. Also, when responses of accurate with missing information declined to inaccurate.

The following are full details of the used AI tools:

- ChatGPT (OpenAI, San Francisco, CA, USA; accessed 31 August 2025);
- Gemini (Google LLC, Mountain View, CA, USA; accessed 31 August 2025);
- Claude (Anthropic PBC, San Francisco, CA, USA; accessed 31 August 2025);
- Perplexity (Perplexity AI, San Francisco, CA, USA; accessed 31 August 2025);
- Consensus (Consensus, Boston, MA, USA; accessed 31 August 2025);
- Poe (Quora, Inc., Mountain View, CA, USA; accessed 31 August 2025).

### 2.3. Statistical Analysis

The precision of each AI tool was expressed as descriptive statistics to summarize the accuracy categorizations of each AI tool across three rounds. The results were then reported as frequency and percentages with corresponding 95% confidence intervals. A 95% confidence interval (CI) for each proportion was calculated using the Wilson method due to the small sample size (*n* = 20 questions). Statistical analysis was applied in SPSS v27 and Stuart–Maxwell test was used for paired categorical comparisons across the three accuracy categories: accurate, accurate with missing information, and inaccurate. The Stuart–Maxwell test was performed for all AI tools for each round separately. Inter-rater reliability between the evaluators was assessed using Cohen’s kappa. A *p*-value < 0.05 was considered statistically significant. To assess the impact of guideline-referenced prompting, responses generated without guideline prompting were compared to responses generated with guideline-referenced prompts for the same questions and AI tools. For this analysis, responses were dichotomized as accurate versus not fully accurate, with accurate responses coded as 1 and not accurate and incomplete responses coded as 0. McNemar’s test was used to compare the proportion of correct responses between the unprompted and the guideline-prompted conditions. A *p* value < 0.05 was considered statistically significant.

## 3. Results

A total of twenty clinical questions were posed to the six AI tools (Supplementary File S1). Responses were under evaluation, and the study was conducted in three separate rounds at six-week intervals. Results were obtained by calculating the mean performance of each category for the included AI tools.

In this study, various AI tools’ accuracy was assessed through three evaluation rounds by providing guideline-based information for diabetes and hypertension management, and distinct performance trends emerged. The following are the steps performed to evaluate all AI tools.

### 3.1. Evaluation of Accuracy for Each Round Independently

- In round 1 (Table 1), ChatGPT and Poe demonstrated the highest accuracy responses at 65% (95% CI: 41.0–83.7), while Consensus lagged at 35% (95% CI: 6.3–59.0). Gemini, Claude, and Perplexity demonstrated medium-level performance, with rates of 55% (95% CI: 32.0–76.2) for Gemini, 60% (95% CI: 36.4–80.0) for Claude, and 55% (95% CI: 32.0–76.2) for Perplexity. In terms of accuracy with missing information, Consensus performed the highest at 10% (95% CI: 29.9–70.1). ChatGPT had the lowest inaccuracy rate at 5% (95% CI: 1.75–33.1).

- Round 2 showed that Claude notably improved to 95% accuracy (95% CI: 73.0–99.7), dominating the results, while Consensus again scored the lowest accuracy performance at 40% (95% CI: 20.0–63.6). In this round, Consensus again demonstrated the highest accuracy for responses with missing information at 45%. In the same round, the inaccuracy rates lessened with both Claude and ChatGPT at 5% (95% CI: 0.26–26.9) (Table 2).

- Round 3 revealed that ChatGPT is the leading AI tool with 85% accuracy (95% CI: 55.7–93.4), while Consensus remained had lowest-level performance at 40% (95% CI: 20.0–63.6). Perplexity demonstrated the highest-level performance with regard to responses containing accurate information with missing data at 30% (95% CI: 12.8–54.3). Inaccuracy was lowest for both Perplexity and Poe at 5% (95% CI: 0.26–26.9), compared to Consensus’s 40% rate (95% CI:20.0–63.6). Overall, Claude demonstrated evident improvement over rounds, while Consensus consistently performed poorly (Table 3).

The Stuart–Maxwell test was used to compare paired categorical responses between AI tools for each round individually across the three accuracy categories: not accurate, accurate, and incomplete. In the first round, no statistically significant differences were observed between the AI tools (all *p* > 0.05). In the second round, overall, statistically significant differences were identified in several pairwise comparisons. Specifically, Claude demonstrated significantly higher accuracy compared with Poe (*p* = 0.0154), Gemini (*p* = 0.0421), Consensus (*p* = 0.0035), and Perplexity (*p* = 0.0302). These findings suggest that Claude provided more accurate responses than the other tools in the second round. In the third round, statistically significant differences were observed between Poe and Consensus (*p* = 0.0279), with Poe demonstrating higher overall accuracy; ChatGPT and Consensus (*p* = 0.0249), with ChatGPT performing better overall accuracy; Claude and Consensus (*p* = 0.0498), with Claude performing better overall accuracy; and Perplexity and Consensus (*p* = 0.0251), with Perplexity demonstrating higher overall accuracy.

### 3.2. Evaluation of Accuracy Trends over Time

**Figure 1:**
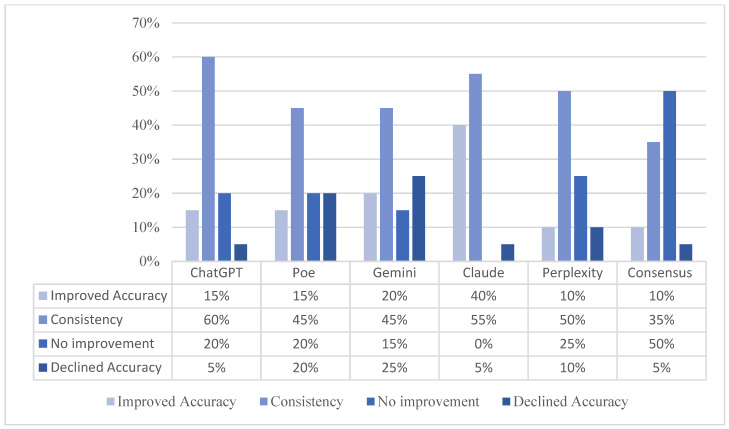
Assessment of performance shift from round 1 to round 2.

The secondary objective of this study focused on evaluating the ability of AI tools to update their responses over time. As accuracy on its own cannot fully reflect the overall performance of these tools, assessing how their responses have changed through multiple rounds reflects a deeper understanding of their reliability and adaptability. By comparing the responses of each tool in three evaluation rounds (Figure 1), we aimed to determine whether the accuracy improved, data upgraded, or consistency was maintained. The outcomes of this objective yield insight into the adaptability and long-term reliability of AI in supporting evidence-based management of diabetes and hypertension (Supplementary File S2).

Figure 1 illustrates the percentage distribution of performance response-shift categories (from “Round 1” to “Round 2”) for the evaluated AI tools. Each color represents one of the four predefined shift categories: improved accuracy, consistency, no improvement, and declined accuracy. Lighter shades indicate favorable or stable response patterns, including improved accuracy and consistency, while darker shades indicate less favorable responses with no improvement or declined accuracy. The percentages shown indicate the proportion of responses assigned to each category for each AI tool.

In the assessment of performance shift from round 1 to round 2 (Figure 1), Claude achieved the greatest improvement in accuracy at 40%, followed by Gemini at 20% and Poe at 15%. Perplexity and Consensus had the lowest achievement of improved accuracy, with both rates at 10%. For consistency, ChatGPT performed highest at 60%, with Claude at 55% and Perplexity at 50%. Poe and Gemini were at 45% and 40%, respectively, while Consensus had the lowest level of performance at 35%. The “no improvement” category showed that Consensus had the highest rate of no improvement at 50%, followed by Perplexity at 25%. Both ChatGPT and Poe registered at 20%, with Gemini at 15%, and Claude exhibited no improvement at 0%. For declined accuracy, Gemini ranked highest at 25%, with Poe at 20% and Perplexity at 10%. ChatGPT, Claude, and Consensus showed a 5% decline.

**Figure 2:**
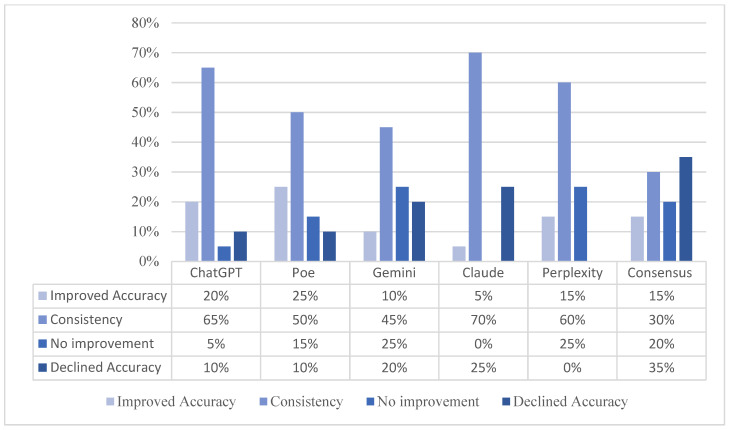
Assessment of performance shift from round 2 to round 3.

Figure 2 illustrates the percentage distribution of performance response-shift categories (from “Round 2” to “Round 3”) for the evaluated AI tools. Each color represents one of the four predefined shift categories: improved accuracy, consistency, no improvement, and declined accuracy. Lighter shades indicate favorable or stable response patterns, including improved accuracy and consistency, while darker shades indicate less favorable responses with no improvement or declined accuracy. The percentages shown indicate the proportion of responses assigned to each category for each AI tool.

In the assessment of performance shift from round 2 to round 3 (Figure 2), Poe improved the most with an accuracy increase of 25%, while ChatGPT followed with 20%. Consensus and Perplexity both achieved a 15% improvement, and Gemini was at 10%, with Claude at 5%. Regarding consistency, Claude led with 70%, followed by ChatGPT at 65%. Perplexity ranked next with 60%, and Poe at 50%, with Gemini at 45% and Consensus at the lowest with 30%. In the “no improvement” metrics, Gemini and Perplexity both had a 25% rate, while Consensus was at 20%. Poe had 15%, whereas Claude displayed no improvement at 0%. Evaluating declined accuracy in round 2, Consensus exhibited 35%, followed by Claude and Gemini. Both ChatGPT and Poe had a 10% decline, while Perplexity showed no decline at 0%.

**Figure 3:**
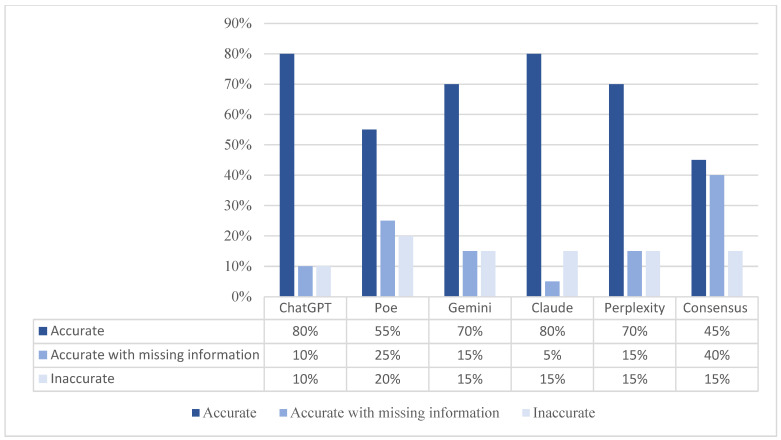
Assessment of the Effect of ADA/AHA referencing on accuracy outcomes.

Figure 3 illustrates the percentage distribution of performance when specific references (ADA and AHA) were given to each AI tool to provide their response. The percentages shown indicate the proportion of responses assigned to each category for each AI tool.

### 3.3. Evaluation of the Impact of ADA/AHA Referencing on Accuracy Outcomes

To consolidate the accuracy of our results, we extended the measurements by providing specific references from the American Diabetes Association (ADA) and the American Heart Association (AHA). As a final stage, we added the statement “According to ADA/AHA” to the questions posed to the AI tools to measure the precision if affected (Figure 3).

The study evaluated the impact of referencing the American Diabetes Association (ADA) and the American Heart Association (AHA) guidelines on AI tool accuracy. Adding “According to ADA/AHA” altered performance metrics: ChatGPT’s performance accuracy reduced from 85% to 80%, while Claude improved from 75% to 80%. Moreover, Perplexity and Gemini improved considerably, with Gemini rising from 55% to 70%. Conversely, Poe’s accuracy dropped from 70% to 55%, while Consensus reduced slightly from 40% to 45%. With respect to accuracy with missing information, Claude demonstrated a decline from 15% to 5%, whereas ChatGPT increased from 5% to 10%. Inaccuracy rates varied, with Claude’s inaccuracies increasing from 10% to 15%, and Perplexity’s rising from 5% to 15%. Consensus notably improved, reducing inaccuracies from 35% to 15%. Overall, guideline-based prompts improved performance for some AI tools while reducing accuracy for others. When evaluating the unprompted and guideline-prompted conditions for all AI tools using McNemar’s test, it did not reveal a statistically significant distinction in the proportion of accurate responses (*p* > 0.05). This implies that the inclusion of guideline references in the prompt did not have a significant effect on the probability of responses.

## 4. Discussion

Patients and medical professionals depend on different online platforms to obtain medical information. Currently, individuals increasingly obtain information from AI tools. The trustworthiness of information produced by AI tools remains a key concern; hence, this study aimed to investigate their reliability, accuracy, and potential for dependable use.

According to the findings, across all rounds, the accuracy was measured by calculating the mean of each AI tool. Claude demonstrated the highest accuracy rate, which may be justified by the fact that Claude obtains the data from more trusted references. Therefore, Claude is more capable of providing guideline-based information; this reduces hallucinations and improves evidence-based output. ChatGPT came second by scoring the lowest rate of inaccurate answers across all rounds, which provides an added advantage. Poe often provides responses similar to ChatGPT, which can be attributed to the fact that Poe is a platform involving different AI models that can access and generate outputs using the ChatGPT model within its platform, while ChatGPT relies solely on its own unified model. Subsequently, Perplexity and Gemini’s tight integration with the Google ecosystem gives them an advantage for Google users [18]. Lastly, Consensus showed the lowest accuracy rate, perhaps due to its summarization-oriented design. Unlike general AI tools, it mainly provides research papers rather than direct information. It is noteworthy that its direct link with traditional tools like PubMed and Google Scholar makes it a better-performing tool for researchers who wish to explore multiple studies rather than seeking immediate guideline-based responses [19].

According to findings, the assessment of performance shifted from round one to round two. We observed a noticeable improvement throughout the rounds; Claude showed the highest improved accuracy rate of 40%. Interestingly, its performance shift from round two to round three dropped to 5% of improved accuracy. Additionally, Gemini demonstrated a decline in performance over time. The underlying reasons for these observations remain unclear. ChatGPT, Poe, Perplexity, and Consensus showed continuous improvement over time, reflecting their adaptive capacity to provide responses that align closely with guideline-based recommendations.

In this study, we aimed to provide standardized references for the AI tools in order to measure differences in their performance. Involving the sentence “According to ADA and AHA guidelines” enabled us to evaluate how guideline-specific information influenced the accuracy of AI responses. Following the addition of guideline-specific references, including ChatGPT and Poe, a decrease in accuracy was demonstrated. In comparison, other tools, such as Gemini, Claude, Perplexity, and Consensus, demonstrated an increase in accuracy, reflecting their improved alignment with the reference guidelines provided.

Previous studies, such as that by Edalati et al., assessed a smaller set and paid versions of AI tools to measure the accuracy compared to medical guidelines [20]; however, we further included six AI tools (ChatGPT, Claude, Gemini, Poe, Perplexity, and Consensus), providing a free and more comprehensive AI tools comparison. Furthermore, previous studies assessed each AI tool only once, using a single evaluation session, while ours assessed their responses over three rounds over a six-week interval to measure consistency and reliability over time. Moreover, we made an additional intervention where we assessed each AI tool under two conditions: first, without providing a specific guideline, while in the second, we provided a specific guideline reference by adding to the questions the phrase “According to ADA/AHA”. Our overall findings show that providing a specific guideline to AI tools might contribute to hallucination and confusion. Finally, unlike previous studies that relied on a single clinical guideline, our study includes four internationally recognized guidelines (ADA, AHA, ESC, and NICE). This wider inclusion of references gives our findings greater comprehensiveness, which indicates that our findings are not biased toward a specific guideline [21,22,23].

The role of AI in clinical practice is increasingly significant, as our findings suggest that AI tools with higher accuracy, such as Claude, could be helpful in clinical management and may enhance efficiency, allowing clinicians to access guideline-based information more effectively. This highlights their potential to assist healthcare providers in their daily practice. However, variation in accuracy across AI tools indicates the need for healthcare providers’ supervision to ensure safety and minimize misinformation risks [24]. The current issue of “accurate with missing information” remains the most critical barrier. In a clinical context, missing a single recommendation can affect a patient’s life. Therefore, the immediate potential of the current generation of AI tools is not suitable for management decision-making, but as a complementary tool [25].

Recent research highlights that hypertension is significantly underdiagnosed and poorly managed worldwide, emphasizing the necessity of precise screening, diagnosis, and follow-up in primary medical care. This underscores the necessity of carefully assessing AI-generated recommendations in hypertension management, since incomplete or incorrect responses may compromise the reliability of these tools in chronic illness education or clinical decision support [26].

One of the important practical applications of AI in clinical practice is emergency department triage. Here, AI-driven technologies could be utilized to aid physicians in prioritizing patients based on urgency and recognizing individuals who require rapid evaluation or intervention. In high-demand clinical environments, such tools may enhance workflow efficiency by enabling early risk classification, enhancing triage uniformity, and optimizing the allocation of limited healthcare resources [27]. A real-world example of AI in clinical practice is the use of autonomous AI systems. These systems are used to screen for diabetic retinopathy by analyzing retinal images and flagging patients who may need to be referred to a specialist [28]. Also, AI has been applied to radiotherapy treatment planning, such as automated plan generation, dose optimization, and more efficient clinical workflows [29]. These examples show the potential utility of AI in a clinical setting and underscore the importance of evaluating the accuracy, completeness, and reliability of the outputs generated by AI prior to widespread clinical use.

Future suggestions for AI developers incorporating clinical guidelines into the AI database could enhance the accuracy of its outcomes. Future studies should investigate how artificial intelligence helps manage therapy and enhance patients’ adherence to medicines, especially for chronic disease like high blood pressure or diabetes, where sticking to treatment really matters. AI systems could identify patients with poor adherence and who are likely to skip doses and then suggest tailored follow-up monitoring. For instance, one study by Perrone and his team assessed patients who had hypertension and were treated with a combination of atorvastatin, perindopril, and amlodipine in a real-world Italian population and highlighted the potential relevance of triple fixed-dose co-formulation for patients receiving these therapies [30]. This example provides an insight into future studies evaluating whether AI can help clinicians identify patients who may benefit from simplified therapeutic regimens, such as fixed-dose combinations, to improve adherence and optimize long-term cardiovascular risk management.

Even if AI can assist healthcare providers in clinical practice, certain limitations need to be taken into consideration. One issue with AI-based clinical decisions is the concern related to transparency, explainability, accountability, bias, and patient safety. AI may generate recommendations, but does not usually present adequate reasoning or justification. This may limit clinicians’ ability to verify the output and apply it correctly to patients in healthcare [31]. Another concern is that the human–AI relationship itself may influence clinical decision-making. Clinicians must understand the role of AI in the decision process and remain responsible for the final decision. Lacking appropriate oversight and explanation, AI tools may create risks such as an overreliance on incorrect recommendations or underuse of potentially helpful outputs [32]. Thus, AI should be considered as a supportive tool that requires expert interpretation, clinical validation, and careful integration into healthcare workflows rather than as a replacement for clinical judgment. Similar concerns have been raised for AI applications in mental health, where AI tools can help with diagnosis, therapy, and monitoring but face limitations in reliability, interpretability, bias, privacy, ethical deployment, and clinical validation. Such parallels indicate that the disconnect between AI capability and clinical readiness is a more general problem across healthcare domains and supports the need for expert oversight and careful evaluation prior to clinical implementation [33].

### Limitations

There are several limitations that should be considered. First, the assessment was limited to a set of twenty clinical questions focusing exclusively on diabetes mellitus and hypertension management. This small sample size restricts the overall performance of the AI tools, which may restrict generalizability. Second, only the free versions of the AI tools were examined; therefore, findings may not reflect the performance of paid versions and the differences between them. Additionally, there was a limitation in measuring the “accuracy with missing information” category, as we were not able to determine whether the “missing” information was essential or non-essential, which may introduce a degree of subjective interpretation. Finally, the study observed notable non-linear shifts in model performance between rounds (e.g., Claude outperforms in round two and subsequently declines in round three for unclear reasons; it was unattainable to identify the variables responsible for these fluctuations).

The study’s results must be regarded with careful consideration because of the limited number of clinical questions used to conduct the assessment. While the questions aimed to evaluate elements of diabetes and hypertension therapy relevant to clinical practice, the restricted sample size may inadequately represent the extensive and complex nature of clinical decision-making in these illnesses. The study exclusively concentrated on the management of diabetes and hypertension; hence, the results may not be applicable to other medical conditions, clinical specialties, or more complicated patient scenarios. Further studies should incorporate a broader and more diverse spectrum of clinical questions across many treatment domains to enhance the assessment of the utility and generalizability of artificial intelligence technologies in healthcare.

## 5. Conclusions

This study assessed the accuracy and reliability of six AI tools (ChatGPT, Claude, Gemini, Poe, Perplexity, and Consensus) in providing guideline-based information for diabetes mellitus and hypertension management. Across all rounds, Claude demonstrated the highest overall performance, followed by ChatGPT with the lowest rate of inaccuracies across all rounds, followed by Poe. When the phrase (“According to ADA/AHA”) was applied, AI tools such as Claude, Gemini, Perplexity, and Consensus showed improvement, while ChatGPT and Poe showed decline, reflecting variability in how AI tools process specific references.

Overall, these findings suggest that AI tools have the potential ability to support healthcare. However, their reliability varied notably across different rounds. This highlights that current AI tools can serve as supportive informational sources but should not replace clinical guideline sources or professional judgment in diabetes mellitus and hypertension management.

## Figures and Tables

**Table 1:** Represents the distribution of response accuracy classification in “Round 1” for all AI tools across the three categories: accurate, accurate with missing information, and inaccurate.

**Round 1**	**AI Tool**	**Accurate n/Total (%)**	**95% CI**	**Accurate with Missing Information n/Total (%)**	**95% CI**	**Inaccurate n/Total (%)**	**95% CI**
	ChatGPT	13 (65%)	41.0–83.7	5 (25%)	9.59–49.4	2 (10%)	1.75–33.1
	Poe	13 (65%)	41.0–83.7	3 (15%)	3.96–38.9	4 (20%)	6.61–44.3
	Gemini	11 (55%)	32.0–76.2	7 (35%)	16.3–59.0	2 (10%)	1.75–33.1
	Claude	12 (60%)	36.4–80.0	6 (30%)	12.8–54.3	2 (10%)	1.75–33.1
	Perplexity	11 (55%)	32.0–76.2	6 (30%)	12.8–54.3	3 (15%)	3.96–38.9
	Consensus	7 (35%)	16.3–59.0	10 (50%)	29.9–70.1	3 (15%)	3.96–38.9

Results are presented as frequencies = *n*, percentage = (%), and corresponding 95% confidence intervals (95% CI) for each AI tool.

**Table 2:** Represents the distribution of response accuracy classification in “Round 2” for all AI tools across the three categories: accurate, accurate with missing information, and inaccurate.

**Round 2**	**AI Tool**	**Accurate n/Total (%)**	**95% CI**	**Accurate with Missing Information n/Total (%)**	**95% CI**	**Inaccurate n/Total (%)**	**95% CI**
	ChatGPT	14 (70%)	45.70–87.2	5 (25%)	9.59–49.4	1 (5%)	0.26–26.9
	Poe	11 (55%)	32.0–76.2	6 (30%)	12.8–54.3	3 (15%)	12.8–54.3
	Gemini	12 (60%)	36.4–80.0	6 (30%)	12.8–54.3	2 (10%)	1.75–33.1
	Claude	19 (95%)	73.0–99.7	0(0%)	___	1 (5%)	0.26–26.9
	Perplexity	12 (60%)	36.4–80.0	4 (20%)	6.6–44.3	4 (20%)	6.61–44.3
	Consensus	8 (40%)	20.0–63.6	9 (45%)	23.8–68.0	3 (15%)	3.96–38.9

Results are presented as frequencies = *n*, percentage = (%), and corresponding 95% confidence intervals (95% CI) for each AI tool.

**Table 3:** Represents the distribution of response accuracy classification in “Round 3” for all AI tools across the three categories: accurate, accurate with missing information, and inaccurate.

**Round 3**	**AI Tool**	**Accurate n/Total (%)**	**95% CI**	**Accurate with Missing Information n/Total (%)**	**95% CI**	**Inaccurate n/Total (%)**	**95% CI**
	**ChatGPT**	17 (85%)	55.7–93.4	1 (5%)	1.75–33.1	2 (10%)	1.75–33.1
	**Poe**	14 (70%)	45.7–87.2	5 (25%)	9.59–49.4	1 (5%)	0.26–26.9
	**Gemini**	11 (55%)	32.0–76.2	5 (25%)	9.59–49.4	4 (20%)	6.61–44.3
	**Claude**	14 (75%)	45.7–87.2	3 (15%)	6.61–44.3	2 (10%)	1.75–33.1
	**Perplexity**	13 (65%)	41.0–83.7	6 (30%)	12.8–54.3	1 (5%)	0.26–26.9
	**Consensus**	8 (40%)	20.0–63.6	4 (20%)	6.6–44.3	8 (40%)	20.0–63.6

Results are presented as frequencies = *n*, percentage = (%), and corresponding 95% confidence intervals (95% CI) for each AI tool.

## Data Availability

The original contributions presented in this study are included in the article/Supplementary Materials. Further inquiries can be directed to the corresponding authors.

## Supplementary Materials

The following supporting information can be downloaded at: https://www.mdpi.com/article/10.3390/jcm15124419/s1; Supplementary File S1: The definition of each response classification and the detailed of the twenty clinical questions that were posed to all AI tools and the responses of each AI tool across all rounds; Supplementary File S2: The detailed description of the twenty clinical questions that were posed to all AI tools and the responses of each AI tool over time.
