# Supplementary material for: Assessing the Accuracy and Precision of Artificial Intelligence for Diabetes Mellitus and Hypertension Management
Source: J Clin Med. 2026 Jun 7;15(12):4419. doi: 10.3390/jcm15124419 (PMC13300920; doi:10.3390/jcm15124419)
Supplement: Supplementary file 1 [file jcm-15-04419-s001.zip › Supplementary File S1.pdf]

**Artificial Intelligence (AI)**	a software applications or platforms that use artificial intelligence algorithms to perform tasks that typically require human intelligence, besides learning and analyzing large amounts of data.
**Diabetes Mellitus**	-
**Hypertension**	-
Accurate	If responses contained complete and guideline-based information or recommendations
Accurate with missing info	If responses were correct but missing information that would be useful for patient care.
Inaccurate	If responses included false or invalid details
Improved accuracy	when a previously inaccurate or accurate with missing information responses became accurate, also responses of inaccurate became accurate with missing information
Consistency	Where responses remained accurate across the previous rounds.
No improvement	When there is no change in response between rounds.
Decline accuracy	When accurate responses decline to accurate with missing information or inaccurate, also responses of accurate with missing information decline to inaccurate.

Round 1						
Questions	Gemini	poe	ChatGPT	Claude	consensus	perplexity
Q1 what is the impact of beta-blockers on glycemic control in diabetic patients with HTN?	Accurate	Accurate	Accurate	Accurate	accurate with missing info	Accurate
Q2 what are the differences in pharmacologic treatment recommendations for DM and HTN in elderly patients?	accurate	accurate with missing info	accurate	accurate	inaccurate	inaccurate
Q3 what are the best exercise recommendations for patients with both DM and HTN?	accurate	accurate	accurate	accurate	accurate	accurate
Q4 What are the first line medications for DM and HTN?	accurate with missing info	inaccurate	accurate with missing info	accurate with missing info	accurate with missing info	accurate
Q5 What are the most common contraindications	Inaccurate	inaccurate	accurate with missing info	accurate with missing info	inaccurate	accurate with missing info

Round 1						
Questions	Gemini	poe	ChatGPT	Claude	consensus	perplexity
for DM and HTN medications?						
Q6 What is the recomended diet modification for DM and HTN?	Accurate	Accurate	Accurate	Accurate with missing information	Accurate with missing information	Accurate with missing information
Q7 What is the role of SGLT2 inhibitors in patients with diabetes and hypertension?	accurate with missing information	accurate (D), Inaccurate (H)	accurate (D) Inaccurate diagnostic criteria of (H)	Accurate with missing	Accurate with missing information	Accurate with missing information
Q8 What are the recommended blood glucose, HbA1c, and blood pressure targets for patients with DM and/or HTN?	accurate	Accurate	Accurate	Accurate with missing information	Accurate with missing information	Accurate with missing information (D)<br><br>Inaccurate (H)
Q9What are the recommendations for complication prevention in patients with DM and HTN?	accurate with missing information (ex; vaccination)	Accurate	accurate with missing information (ex; vaccination)	Accurate	Accurate with missing information	Accurate
Q10 what are the guidelines recommend to assessing a patient's risk for	accurate	Accurate	Accurate	Accurate	Inaccurate	Inaccurate

Round 1						
Questions	Gemini	poe	ChatGPT	Claude	consensus	perplexity
developing DM and/or HTN?						
Q11What are the recommended frequencies for monitoring blood glucose, HbA1c, and blood pressure in patients with DM and/or HTN?	Inaccurate	Accurate with missing information	Accurate with missing information	Accurate	Accurate with missing information	Accurate with missing information
Q12 what are the medications recommended for hypertension and DM in pregnant persons?	Accurate with missing information	Accurate	Accurate	Accurate	Accurate with missing information	Accurate with missing information
Q13 What are the guidelines address the management of DM and HTN medications in	Accurate with missing information	Accurate	Accurate	Accurate with missing information	Accurate	Accurate

Round 1						
Questions	Gemini	poe	ChatGPT	Claude	consensus	perplexity
the context of chronic kidney disease ?						
Q14 What is the main treatment of what is the main treatment of DKA?	Accurate	Accurate	Accurate	Inaccurate	Accurate with missing information	Accurate
Q15 What is the main non-pharmacotherapy to treat HTN?	Accurate with missing information	Inaccurate	Inaccurate	Inaccurate	Accurate with missing information	Accurate with missing information
Q16Which antihypertensive classes are preferred in patients with diabetes and evidence of nephropathy?	Accurate with missing information	Accurate with missing information	Accurate with missing information	Accurate	Accurate	Accurate
Q17 What is the first choice treatment in	Accurate	Accurat	Accurat	Accurat	Accurat	Accurat

Round 1						
Questions	Gemini	poe	ChatGPT	Claude	consensus	perplexity
patients with DM2 ?						
Q18 what is the treatment of choice in Gestational diabetes?	Accurat	Accurat	Accurat	Accurat	Accurat	Accurat
Q19 What is the best diet for patients with uncontrolled type 2 diabetes mellitus (DM2)?	Accurate	Accurate	Accurate	Accurate	Accurate	Accurate
Q20 What is the preferred antihypertensive therapy for patients with diabetes and proteinuria?	Accurate	Accurate	Accurate	Accurate	Accurate	Accurate

Round 2						
Questions	gimini	poe	chatgpt	claude	consensus	preplexity
Q1 what is the impact of beta-blockers on glycemic control	accurate	accurate	accurate	accurate	Accurate with missing info	accurate

Round 2						
Questions	gimini	poe	chatgpt	claude	consensus	preplexity
in diabetic patients with HTN?						
Q2 what are the differences in pharmacologic treatment recommendations for DM and HTN in elderly patients?	accurate	accurate	accurate	accurate	accurate with missing info	inaccurate
Q3 what are the best exercise recommendations for patients with both DM and HTN?	accurate	accurate	accurate	accurate	accurate	accurate
Q4 What are the first line medications for DM and HTN?	accurate	accurate	accurate	accurate	inaccurate	accurate
Q5 What are the most common contraindications for DM and HTN medications?	accurate with missing info(H),accurate(D)	accurate with missing info	accurate with missing info,	accurate	inaccurate	accurate with missing info

Round 2						
Questions	gimini	poe	chatgpt	claude	consensus	preplexity
Q6 What is the recomended diet modification for DM and HTN?	accurate with missing information	accurate with missing information	accurate with missing information	Accurate	Accurate with missing information	Accurate with missing information
Q7 What is the role of SGLT2 inhibitors in patients with diabetes and hypertension?	accurate (D), Accurate with missing information (H)	accurate (D) Inaccurate (H)	accurate with missing information	Accurate	Accurate with missing information	Accurate with missing information (D), Inaccurate (H)
Q8 What are the recommended blood glucose, HbA1c, and blood pressure targets for patients with DM and/or HTN?	accurate	accurate (D) missing information (H) Accurate with	Accurate	Accurate (D) Inaccurate (H)	Accurate with missing information	Accurate with missing information (D), Inaccurate (H)
Q9What are the recommendations for complication prevention in patients with DM and HTN?	accurate with missing information	accurate with missing information	accurate with missing info	Accurate	Accurate with missing information	Accurate
Q10 what are the guidelines recommend to assessing a	accurate with missing information	inaccurate	accurate	Accurate	Inaccurate	Accurate with missing information

Round 2						
Questions	gimini	poe	chatgpt	claude	consensus	preplexity
patient's risk for developing DM and/or HTN?						
Q11What are the recommended frequencies for monitoring blood glucose, HbA1c, and blood pressure in patients with DM and/or HTN?	Accurate with missing information	Accurate with missing information	Accurate with missing information	Accurate	Accurate	Accurate with missing information
Q12 what are the medications recommended for hypertension and DM in pregnant persons?	Accurate	Accurate	Accurate	Accurate	Accurate with missing information	Accurate

Round 2						
Questions	gimini	poe	chatgpt	claude	consensus	preplexity
Q13 What are the guidelines address the management of DM and HTN medications in the context of chronic kidney disease ?	Inaccurate	Accurate	Accurate	Accurate	Accurate	Accurate
Q14 What is the main treatment of what is the main treatment of DKA?	Accurate	Accurate	Accurate	Accurate	Accurate with missing information	Inaccurate
Q15 What is the main non-pharmacotherapy to treat HTN?	Inaccurate	Inaccurate	Inaccurate	Accurate	Accurate with missing information	Accurate#
Q16 Which antihypertensive classes are preferred in patients with diabetes and	Accuaccrate	Accurate with missing information	Accurate	Accurate	Accurate	Accurate

Round 2						
Questions	gimini	poe	chatgpt	claude	consensus	preplexity
evidence of nephropathy?						
Q17 What is the first choice treatment in patients with DM2 ?	Accurate	Accurate	Accurate	Accurate	Accurate	Accurate
Q18 what is the treatment of choice in Gestational diabetes?	Accurate	Accurate	Accurate	Accurate	Accurate	Accurate
Q19 What is the best diet for patients with uncontrolled type 2 diabetes mellitus (DM2)?	Accurate	Accurate	Accurate	Accurate	Accurate	Accurate
Q20 What is the preferred antihypertensive therapy for patients with diabetes and proteinuria?	Accurate	Accurate	Accurate	Accurate	Accurate	Accurate

Round 3						
Questions	gimini	poe	chatgpt	claude	consensus	preplexity
Q1 what is the impact of beta-blockers on glycemic control in diabetic	accurate	accurate	accurate	accurate	Accurate with missing info	accurate

Round 3						
Questions	gimini	poe	chatgpt	claude	consensus	preplexity
patients with HTN?						
Q2 what are the differences in pharmacologic treatment recommendations for DM and HTN in elderly patients?	Inaccurate	accurate	accurate	accurate	Inaccurate	accurate with missing info
Q3 what are the best exercise recommendations for patients with both DM and HTN?	accurate	accurate	accurate	accurate	accurate	accurate
Q4 What are the first line medications for DM and HTN?	accurate	accurate with missing info	accurate	accurate	inaccurate	accurate
Q5 What are the most common contraindications for DM and HTN medications?	accurate	accurate	accurate	accurate	Inaccurate	accurate with missing info

Round 3						
Questions	gimini	poe	chatgpt	claude	consensus	preplexity
Q6 What is the recomended diet modification for DM and HTN?	Accurate with missing information	Accurate	Accurate	Accurate with missing information	Accurate with missing information	Accurate with missing information
Q7 What is the role of SGLT2 inhibitors in patients with diabetes and hypertension?	Inaccurate	Accurate with missing information	Inaccurate	Accurate	Accurate	Accurate
Q8 What are the recommended blood glucose, HbA1c, and blood pressure targets for patients with DM and/or HTN?	Inaccurate	Accurate with missing information	Accurate	Accurate	Inaccurate	Accurate with missing information
Q9What are the recommendations for complication prevention in patients with DM and HTN?	Accurate with missing information	Accurate with missing information	Accurate	Accurate with missing information	Accurate	Accurate
Q10 what are the guidelines recommend to assessing a	Accurate with missing information	Accurate	Accurate	Accurate	Accurate with missing information	Accurate with missing information

Round 3						
Questions	gimini	poe	chatgpt	claude	consensus	preplexity
patient's risk for developing DM and/or HTN?						
Q11What are the recommended frequencies for monitoring blood glucose, HbA1c, and blood pressure in patients with DM and/or HTN?	Accurate with missing information	Accurate with missing information	Accurate	Accurate with missing information	Accurate with missing information	Accurate with missing information
Q12 what are the medications recommended for hypertension and DM in pregnant persons?	Accurate	Accurate	Accurate	Accurate	Inaccurate	Accurate
Q13 What are the guidelines address the management of DM and HTN medications in the	Inaccurate	Accurate	Accurate	Accurate	Inaccurate	Accurate

.

Round 3						
Questions	gimini	poe	chatgpt	claude	consensus	preplexity
context of chronic kidney disease ?						
Q14 What is the main treatment of what is the main treatment of DKA?	Accurate with missing information	Inaccurate	Accurate with missing information	Inaccurate	Inaccurate	Inaccurate
Q15 What is the main non-pharmacotherapy to treat HTN?	Accurate	Accurate	Inaccurate	Inaccurate	Inaccurate	Accurate
Q16 Which antihypertensive classes are preferred in patients with diabetes and evidence of nephropathy?	Accurate	Accurate	Accurate	Accurate	Accurate	Accurate
Q17 What is the first choice treatment in	Accurate	Accurate	Accurate	Accurate	Accurate	Accurate

Round 3						
Questions	gimini	poe	chatgpt	claude	consensus	preplexity
patients with DM2 ?						
Q18 what is the treatment of choice in Gestational diabetes?	Accurate	Accurate	Accurate	Accurate	Accurate	Accurate
Q19 What is the best diet for patients with uncontrolled type 2 diabetes mellitus (DM2)?	Accurate	Accurate	Accurate	Accurate	Accurate	Accurate
Q20 What is the preferred antihypertensive therapy for patients with diabetes and proteinuria?	Accurate	Accurate	Accurate	Accurate	Accurate	Accurate

Adding according to..						
Questions	gimini	poe	chatgpt	claude	consensus	preplexity
Q1 what is the impact of beta-blockers on glycemic control in diabetic patients with HTN?	accurate	inaccurate	accurate	accurate	Accurate with missing info	accurate
Q2 what are the differences in pharmacologic treatment recommendations for DM and HTN in elderly patients?	accurate	accurate	accurate	inaccurate	accurate	inaccurate
Q3 what are the best exercise recommendations for patients with both DM and HTN?	accurate	accurate	accurate	accurate	accurate	accurate
Q4 What are the first line medications for DM and HTN?	inaccurate	accurate with missing info	accurate	accurate	inaccurate	accurate
Q5 What are the most common	accurate	accurate	accurate	accurate	inaccurate	inaccurate

Adding according to..						
Questions	gimini	poe	chatgpt	claude	consensus	preplexity
contraindications for DM and HTN medications?						
Q6 What is the recomended diet modification for DM and HTN?	accurateo	accurate	accurate	accurate	Accurate with missing information	accurate
Q7 What is the role of SGLT2 inhibitors in patients with diabetes and hypertension?	accurate	Accurate with missing information	Accurate	Accurate	Accurate with missing information	Accurate with missing information
Q8 What are the recommended blood glucose, HbA1c, and blood pressure targets for patients with DM and/or HTN?	Accurate	Accurate with missing information	Accurate	Accurate	Accurate	Accurate
Q9What are the recommendations for complication prevention in patients with DM and HTN?	Accurate with missing information	Inaccurate	accurate	accurate	Accurate with missing information	Accurate with missing information

Adding according to..						
Questions	gimini	poe	chatgpt	claude	consensus	preplexity
Q10 what are the guidelines recommend to assessing a patient's risk for developing DM and/or HTN?	inaccurate	inaccurate	accurate	accurate	Accurate with missing information	Accurate with missing information
Q11What are the recommended frequencies for monitoring blood glucose, HbA1c, and blood pressure in patients with DM and/or HTN?	Accurate	Accurate with missing information	Accurate with missing information	Accurate with missing information	Accurate with missing information	Accurate
Q12 what are the medications recommended for hypertension and DM in pregnant persons?	Accurate	Accurate	Accurate	Accurate	Accurate with missing information	Accurate
Q13 What are the guidelines address the management	Accurate with missing information	Accurate	Accurate with missing information	Accurate	Accurate	Accurate

Adding according to..						
Questions	gimini	poe	chatgpt	claude	consensus	preplexity
of DM and HTN medications in the context of chronic kidney disease ?						
Q14 What is the main treatment of what is the main treatment of DKA?	Accurate with missing information	Accurate with missing information	Inaccurate	Inaccurate	Accurate with missing information	Accurate
Q15 What is the main non-pharmacotherapy to treat HTN?	Inaccurate	Inaccurate	Inaccurate	Inaccurate	Inaccurate	Inaccurate
Q16 Which antihypertensive classes are preferred in patients with diabetes and evidence of nephropathy?	accurate	accurate	accurate	accurate	accurate	accurate

Adding according to..						
Questions	gimini	poe	chatgpt	claude	consensus	preplexity
Q17 What is the first choice treatment in patients with DM2 ?	accurate	accurate	accurate	accurate	accurate	accurate
Q18 what is the treatment of choice in Gestational diabetes?	accurate	accurate	accurate	accurate	accurate	accurate
Q19 What is the best diet for patients with uncontrolled type 2 diabetes mellitus (DM2)?	accurate	accurate	accurate	accurate	accurate	accurate
Q20 What is the preferred antihypertensive therapy for patients with diabetes and proteinuria?	accurate	accurate	accurate	accurate	accurate	accurate
