# Supplementary material for: Assessing the Accuracy and Precision of Artificial Intelligence for Diabetes Mellitus and Hypertension Management
Source: J Clin Med. 2026 Jun 7;15(12):4419. doi: 10.3390/jcm15124419 (PMC13300920; doi:10.3390/jcm15124419)
Supplement: Supplementary file 1 [file jcm-15-04419-s001.zip › Supplementary File S2.pdf]

R1 → R2 (ChatGPT)

Question	Round 1	Round 2	Improved Accuracy	Consistency	No Improvement	Declined Accuracy
1- What is the impact of beta-blockers on glycemic control in diabetic patients with HTN?	Accurate	Accurate		●		
2-What are the differences in pharmacologic treatment recommendations for DM and HTN in elderly patients?	Accurate	Accurate		●		
3-What are the best exercise recommendations for patients with both DM and HTN?	Accurate	Accurate		●		
4-What are the first line medications for DM and HTN?	Accurate with missing information	Accurate	●			
5-What are the most common contraindications for DM and HTN medications?	Accurate with missing information	Accurate with missing information			●	
6- What is the recomended diet modification for DM and HTN?	Accurate	Accurate with missing information				●
7- What is the recomended diet modification for DM and HTN?	Inaccurate	Accurate with missing information				
8- What is the recomended diet modification for DM and HTN?	Accurate	Accurate		●		

9- What is the recomended diet modification for DM and HTN?	Accurate with missing information	Accurate with missing information			●	
10- What is the recomended diet modification for DM and HTN?	Accurate	Accurate		●		
Q11What are the recommended frequencies for monitoring blood glucose, HbA1c, and blood pressure in patients with DM and/or HTN?	Accurate with missing information	Accurate with missing information			●	
Q12 what are the medications recommended for hypertension and DM in pregnant persons?	Accurate	Accurate		●		
Q13 What are the guidelines address the management of DM and HTN medications in the context of chronic kidney disease ?	Accurate	Accurate		●		
Q14 What is the main treatment of what is the main treatment of DKA?	Accurate	Accurate		●		
Q15 What is the main non-pharmacotherapy to treat HTN?	Inaccurate	Inaccurate			●	
16.Which antihypertensive classes are preferred in patients with diabetes and evidence of nephropathy?	Accurate with missing information	Accurate	●			
17.What is the first choice treatment in patients with DM2 ?	Accurate	Accurate		●		
18.what is the treatment of choice in Gestational diabetes? What is the best diet for patients with uncontrolled type 2 diabetes mellitus (DM2)?	Accurate	Accurate		●		
19.What is the preferred antihypertensive therapy for patients with diabetes and proteinuria?	Accurate	Accurate		●		

Q20. What is the preferred antihypertensive therapy for patients with diabetes and proteinuria?	Accurate	Accurate				

R1 → R2 (Gemini)						
Question	Round 1	Round 2	Improved Accuracy	Consistency	No Improvement	Declined Accuracy
1- What is the impact of beta-blockers on glycemic control in diabetic patients with HTN?	Accurate	Accurate		●		
2-What are the differences in pharmacologic treatment recommendations for DM and HTN in elderly patients?	Accurate	Accurate		●		
3-What are the best exercise recommendations for patients with both DM and HTN?	Accurate	Accurate		●		
4-What are the first line medications for DM and HTN?	Accurate with missing data	Accurate	●			
5-What are the most common contraindications for DM and HTN medications?	Accurate with missing information	Accurate with missing information			●	
6- What is the recomended diet modification for DM and HTN?	Accurate	Accurate with missing information				●
7- What is the recomended diet modification for DM and HTN?	Accurate with missing information	Accurate with missing information			●	
8- What is the recomended diet modification for DM and HTN?	Accurate	Accurate with missing information				●

9- What is the recomended diet modification for DM and HTN?	Accurate with missing information	Accurate with missing information			●	
10- What is the recomended diet modification for DM and HTN?	Accurate	Accurate with missing information				●
Q11What are the recommended frequencies for monitoring blood glucose, HbA1c, and blood pressure in patients with DM and/or HTN?	Inaccurate	Accurate with missing information	●			
Q12 what are the medications recommended for hypertension and DM in pregnant persons?	Accurate with missing information	Accurate	●			
Q13 What are the guidelines address the management of DM and HTN medications in the context of chronic kidney disease ?	Accurate with missing information	Inaccurate				●
Q14 What is the main treatment of what is the main treatment of DKA?	Accurate	Accurate		●		
Q15 What is the main non-pharmacotherapy to treat HTN?	Accurate with missing information	Inaccurate				●
16.Which antihypertensive classes are preferred in patients with diabetes and evidence of nephropathy?	Accurate with missing information	Accurate	●			
17.What is the first choice treatment in patients with DM2 ?	Accurate	Accurate		●		
18.what is the treatment of choice in Gestational diabetes? What is the best diet for patients with uncontrolled type 2 diabetes mellitus (DM2)?	Accurate	Accurate		●		
19.What is the preferred antihypertensive therapy for patients with diabetes and proteinuria?	Accurate	Accurate		●		

Q20. What is the preferred antihypertensive therapy for patients with diabetes and proteinuria?	Accurate	Accurate				

**R1 → R2 (Poe)**						
Question	Round 1	Round 2	Improved Accuracy	Consistency	No Improvement	Declined Accuracy
1- What is the impact of beta-blockers on glycemic control in diabetic patients with HTN?	Accurate	Accurate		●		
2-What are the differences in pharmacologic treatment recommendations for DM and HTN in elderly patients?	Accurate with missing information	Accurate	●			
3-What are the best exercise recommendations for patients with both DM and HTN?	Accurate	Accurate		●		
4-What are the first line medications for DM and HTN?	Inaccurate	Accurate	●			
5-What are the most common contraindications for DM and HTN medications?	Inaccurate	Accurate with missing information	●			
6- What is the recomended diet modification for DM and HTN?	Accurate	Accurate with missing information				
7- What is the recomended diet modification for DM and HTN?	Inaccurate	Inaccurate			●	●
8- What is the recomended diet modification for DM and HTN?	Accurate	Accurate with missing information				●
9- What is the recomended diet modification for DM and HTN?	Accurate	Accurate with missing information				●
10- What is the recomended diet modification for DM and HTN?	Accurate	Inaccurate				●

Q11What are the recommended frequencies for monitoring blood glucose, HbA1c, and blood pressure in patients with DM and/or HTN?	Accurate with missing information	Accurate with missing information			●	
Q12 what are the medications recommended for hypertension and DM in pregnant persons?	Accurate	Accurate		●		
Q13 What are the guidelines address the management of DM and HTN medications in the context of chronic kidney disease ?	Accurate	Accurate		●		
Q14 What is the main treatment of what is the main treatment of DKA?	Accurate	Accurate		●		
Q15 What is the main non-pharmacotherapy to treat HTN?	Inaccurate	Inaccurate			●	
16.Which antihypertensive classes are preferred in patients with diabetes and evidence of nephropathy?	Accurate with missing information	Accurate with missing information			●	
17.What is the first choice treatment in patients with DM2 ?	Accurate	Accurate		●		
18.what is the treatment of choice in Gestational diabetes? What is the best diet for patients with uncontrolled type 2 diabetes mellitus (DM2)?	Accurate	Accurate		●		
19.What is the preferred antihypertensive therapy for patients with diabetes and proteinuria?	Accurate	Accurate		●		
Q20. What is the preferred antihypertensive therapy for patients with diabetes and proteinuria?	Accurate	Accurate		●		

R1 → R2 (Claude)						
Question	Round 1	Round 2	Improved Accuracy	Consistency	No Improvement	Declined Accuracy
1- What is the impact of beta-blockers on glycemic control in diabetic patients with HTN?	Accurate	Accurate		●		
2-What are the differences in pharmacologic treatment recommendations for DM and HTN in elderly patients?	Accurate	Accurate		●		
3-What are the best exercise recommendations for patients with both DM and HTN?	Accurate	Accurate		●		
4-What are the first line medications for DM and HTN?	Accurate with missing information	Accurate	●			
5-What are the most common contraindications for DM and HTN medications?	Accurate with missing information	Accurate	●			
6- What is the recomended diet modification for DM and HTN?	Accurate with missing information	Accurate	●			
7- What is the recomended diet modification for DM and HTN?	Accurate with missing information	Accurate	●			
8- What is the recomended diet modification for DM and HTN?	Accurate with missing information	Inaccurate				●
9- What is the recomended diet modification for DM and HTN?	Accurate	Accurate		●		

10- What is the recomended diet modification for DM and HTN?	Accurate with missing information	Accurate	●			
Q11What are the recommended frequencies for monitoring blood glucose, HbA1c, and blood pressure in patients with DM and/or HTN?	Accurate	Accurate		●		
Q12 what are the medications recommended for hypertension and DM in pregnant persons?	Accurate	Accurate		●		
Q13 What are the guidelines address the management of DM and HTN medications in the context of chronic kidney disease ?	Accurate with missing information	Accurate	●			
Q14 What is the main treatment of what is the main treatment of DKA?	Inaccurate	Accurate	●			
Q15 What is the main non-pharmacotherapy to treat HTN?	Inaccurate	Accurate	●			
16.Which antihypertensive classes are preferred in patients with diabetes and evidence of nephropathy?	Accurate	Accurate		●		
17.What is the first choice treatment in patients with DM2 ?	Accurate	Accurate		●		
18.what is the treatment of choice in Gestational diabetes?<br>What is the best diet for patients with uncontrolled type 2 diabetes mellitus (DM2)?	Accurate	Accurate		●		
19.What is the preferred antihypertensive therapy for patients with diabetes and proteinuria?	Accurate	Accurate		●		

Q20. What is the preferred antihypertensive therapy for patients with diabetes and proteinuria?	Accurate	Accurate				

**R1 → R2 (perplexity)**						
Question	Round 1	Round 2	Improved Accuracy	Consistency	No Improvement	Declined Accuracy
1- What is the impact of beta-blockers on glycemic control in diabetic patients with HTN?	Accurate	Accurate		●		
2-What are the differences in pharmacologic treatment recommendations for DM and HTN in elderly patients?	Inaccurate	Inaccurate			●	
3-What are the best exercise recommendations for patients with both DM and HTN?	Accurate	Accurate		●		
4-What are the first line medications for DM and HTN?	Accurate	Accurate		●		
5-What are the most common contraindications for DM and HTN medications?	Accurate with missing information	Accurate with missing information			●	
6- What is the recomended diet modification for DM and HTN?	Accurate with missing information	Accurate with missing information			●	
7- What is the recomended diet modification for DM and HTN?	Accurate with missing information	Inaccurate				●
8- What is the recomended diet modification for DM and HTN?	Inaccurate	Inaccurate			●	
9- What is the recomended diet modification for DM and HTN?	Accurate	Accurate		●		
10- What is the recomended diet modification for DM and HTN?	Inaccurate	Accurate with missing information				

Q11What are the recommended frequencies for monitoring blood glucose, HbA1c, and blood pressure in patients with DM and/or HTN?	Accurate with missing information	Accurate with missing information			●	
Q12 what are the medications recommended for hypertension and DM in pregnant persons?	Accurate with missing information	Accurate	●			
Q13 What are the guidelines address the management of DM and HTN medications in the context of chronic kidney disease ?	Accurate	Accurate		●		
Q14 What is the main treatment of what is the main treatment of DKA?	Accurate	Inaccurate				●
Q15 What is the main non-pharmacotherapy to treat HTN?	Accurate with missing information	Accurate	●			
16.Which antihypertensive classes are preferred in patients with diabetes and evidence of nephropathy?	Accurate	Accurate		●		
17.What is the first choice treatment in patients with DM2 ?	Accurate	Accurate		●		
18.what is the treatment of choice in Gestational diabetes? What is the best diet for patients with uncontrolled type 2 diabetes mellitus (DM2)?	Accurate	Accurate		●		
19.What is the preferred antihypertensive therapy for patients with diabetes and proteinuria?	Accurate	Accurate		●		
Q20. What is the preferred antihypertensive therapy for patients with diabetes and proteinuria?	Accurate	Accurate		●		

**R1 → R2 (consensus)**						
Question	Round 1	Round 2	Improved Accuracy	Consistency	No Improvement	Declined Accuracy
1- What is the impact of beta-blockers on glycemic control in diabetic patients with HTN?	Accurate with missing information	Accurate with missing information			●	
2-What are the differences in pharmacologic treatment recommendations for DM and HTN in elderly patients?	Inaccurate	Accurate with missing information	●			
3-What are the best exercise recommendations for patients with both DM and HTN?	Accurate	Accurate		●		
4-What are the first line medications for DM and HTN?	Accurate with missing information	Inaccurate				●
5-What are the most common contraindications for DM and HTN medications?	Inaccurate	Inaccurate			●	
6- What is the recomended diet modification for DM and HTN?	Accurate with missing information	Accurate with missing information			●	
7- What is the recomended diet modification for DM and HTN?	Accurate with missing information	Accurate with missing information			●	
8- What is the recomended diet modification for DM and HTN?	Accurate with missing information	Accurate with missing information			●	
9- What is the recomended diet modification for DM and HTN?	Accurate with missing information	Accurate with missing information			●	

10- What is the recomended diet modification for DM and HTN?	Inaccurate	Inaccurate			●	
Q11What are the recommended frequencies for monitoring blood glucose, HbA1c, and blood pressure in patients with DM and/or HTN?	Accurate with missing information	Accurate	●			
Q12 what are the medications recommended for hypertension and DM in pregnant persons?	Accurate with missing information	Accurate with missing information			●	
Q13 What are the guidelines address the management of DM and HTN medications in the context of chronic kidney disease ?	Accurate	Accurate		●		
Q14 What is the main treatment of what is the main treatment of DKA?	Accurate with missing information	Accurate with missing information			●	
Q15 What is the main non-pharmacotherapy to treat HTN?	Accurate with missing information	Accurate with missing information			●	
16.Which antihypertensive classes are preferred in patients with diabetes and evidence of nephropathy?	Accurate	Accurate		●		
17.What is the first choice treatment in patients with DM2 ?	Accurate	Accurate		●		
18.what is the treatment of choice in Gestational diabetes? What is the best diet for patients with uncontrolled type 2 diabetes mellitus (DM2)?	Accurate	Accurate		●		
19.What is the preferred antihypertensive therapy for	Accurate	Accurate		●		

patients with diabetes and proteinuria?						
Q20. What is the preferred antihypertensive therapy for patients with diabetes and proteinuria?	Accurate	Accurate		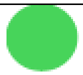		

R2 → **R3 (ChatGPT)**						
Question	Round 2	Round 3	Improved Accuracy	Consistency	No Improvement	Declined Accuracy
1- What is the impact of beta-blockers on glycemic control in diabetic patients with HTN?	Accurate	Accurate		●		
2-What are the differences in pharmacologic treatment recommendations for DM and HTN in elderly patients?	Accurate	Accurate		●		
3-What are the best exercise recommendations for patients with both DM and HTN?	Accurate	Accurate		●		
4-What are the first line medications for DM and HTN?	Accurate	Accurate		●		
5-What are the most common contraindications for DM and HTN medications?	Accurate with missing information	Accurate	●			
6- What is the recomended diet modification for DM and HTN?	Accurate with missing information	Accurate	●			
7- What is the recomended diet modification for DM and HTN?	Accurate with missing information	Inaccurate				●
8- What is the recomended diet modification for DM and HTN?	Accurate	Accurate		●		
9- What is the recomended diet modification for DM and HTN?	Accurate with missing information	Accurate	●			

10- What is the recomended diet modification for DM and HTN?	Accurate	Accurate		●		
Q11What are the recommended frequencies for monitoring blood glucose, HbA1c, and blood pressure in patients with DM and/or HTN?	Accurate with missing information	Accurate	●			
Q12 what are the medications recommended for hypertension and DM in pregnant persons?	Accurate	Accurate		●		
Q13 What are the guidelines address the management of DM and HTN medications in the context of chronic kidney disease ?	Accurate	Accurate		●		
Q14 What is the main treatment of what is the main treatment of DKA?	Accurate	Accurate with missing information				●
Q15 What is the main non-pharmacotherapy to treat HTN?	Inaccurate	Inaccurate			●	
16.Which antihypertensive classes are preferred in patients with diabetes and evidence of nephropathy?	Accurate	Accurate		●		
17.What is the first choice treatment in patients with DM2 ?	Accurate	Accurate		●		
18.what is the treatment of choice in Gestational diabetes? What is the best diet for patients with uncontrolled type 2 diabetes mellitus (DM2)?	Accurate	Accurate		●		
19.What is the preferred antihypertensive therapy for patients with diabetes and proteinuria?	Accurate	Accurate		●		

Q20. What is the preferred antihypertensive therapy for patients with diabetes and proteinuria?	Accurate	Accurate		●		

R2 → R3 ((Gemini)						
Question	Round 2	Round 3	Improved Accuracy	Consistency	No Improvement	Declined Accuracy
1- What is the impact of beta-blockers on glycemic control in diabetic patients with HTN?	Accurate	Accurate		●		
2-What are the differences in pharmacologic treatment recommendations for DM and HTN in elderly patients?	Accurate	Inaccurate				●
3-What are the best exercise recommendations for patients with both DM and HTN?	Accurate	Accurate		●		
4-What are the first line medications for DM and HTN?	Accurate	Accurate		●		
5-What are the most common contraindications for DM and HTN medications?	Accurate with missing information	Accurate	●			
6- What is the recomended diet modification for DM and HTN?	Accurate with missing information	Accurate with missing information			●	
7- What is the recomended diet modification for DM and HTN?	Accurate with missing information	Inaccurate				●
8- What is the recomended diet modification for DM and HTN?	Accurate with missing information	Inaccurate				●

9- What is the recomended diet modification for DM and HTN?	Accurate with missing information	Accurate with missing information			●	
10- What is the recomended diet modification for DM and HTN?	Accurate with missing information	Accurate with missing information			●	
Q11What are the recommended frequencies for monitoring blood glucose, HbA1c, and blood pressure in patients with DM and/or HTN?	Accurate with missing information	Accurate with missing information			●	
Q12 what are the medications recommended for hypertension and DM in pregnant persons?	Accurate	Accurate		●		
Q13 What are the guidelines address the management of DM and HTN medications in the context of chronic kidney disease ?	Inaccurate	Inaccurate			●	
Q14 What is the main treatment of what is the main treatment of DKA?	Accurate	Accurate with missing information				●
Q15 What is the main non-pharmacotherapy to treat HTN?	Inaccurate	Accurate	●			
16.Which antihypertensive classes are preferred in patients with diabetes and evidence of nephropathy?	Accurate	Accurate		●		
17.What is the first choice treatment in patients with DM2 ?	Accurate	Accurate		●		
18.what is the treatment of choice in Gestational diabetes? What is the best diet for patients with uncontrolled type 2 diabetes mellitus (DM2)?	Accurate	Accurate		●		
19.What is the preferred antihypertensive therapy for patients with diabetes and proteinuria?	Accurate	Accurate		●		

Q20. What is the preferred antihypertensive therapy for patients with diabetes and proteinuria?	Accurate	Accurate		●		

R2 → R3 (Poe)						
Question	Round 2	Round 3	Improved Accuracy	Consistency	No Improvement	Declined Accuracy
1- What is the impact of beta-blockers on glycemic control in diabetic patients with HTN?	Accurate	Accurate		●		
2-What are the differences in pharmacologic treatment recommendations for DM and HTN in elderly patients?	Accurate	Accurate		●		
3-What are the best exercise recommendations for patients with both DM and HTN?	Accurate	Accurate		●		
4-What are the first line medications for DM and HTN?	Accurate	Accurate with missing information				●
5-What are the most common contraindications for DM and HTN medications?	Accurate with missing information	Accurate	●			
6- What is the recomended diet modification for DM and HTN?	Accurate with missing information	Accurate	●			
7- What is the recomended diet modification for DM and HTN?	Inaccurate	Accurate with missing information	●			
8- What is the recomended diet modification for DM and HTN?	Accurate with missing information	Accurate with missing information			●	
9- What is the recomended diet modification for DM and HTN?	Accurate with missing information	Accurate with missing information			●	

10- What is the recomended diet modification for DM and HTN?	Inaccurate	Accurate	●			
Q11What are the recommended frequencies for monitoring blood glucose, HbA1c, and blood pressure in patients with DM and/or HTN?	Accurate with missing information	Accurate with missing information			●	
Q12 what are the medications recommended for hypertension and DM in pregnant persons?	Accurate	Accurate		●		
Q13 What are the guidelines address the management of DM and HTN medications in the context of chronic kidney disease ?	Accurate	Accurate		●		
Q14 What is the main treatment of what is the main treatment of DKA?	Accurate	Inaccurate				●
Q15 What is the main non-pharmacotherapy to treat HTN?	Inaccurate	Accurate	●			
16.Which antihypertensive classes are preferred in patients with diabetes and evidence of nephropathy?	Accurate	Accurate		●		
17.What is the first choice treatment in patients with DM2 ?	Accurate	Accurate		●		
18.what is the treatment of choice in Gestational diabetes?<br>What is the best diet for patients with uncontrolled type 2 diabetes mellitus (DM2)?	Accurate	Accurate		●		
19.What is the preferred antihypertensive therapy for patients with diabetes and proteinuria?	Accurate	Accurate		●		

Q20. What is the preferred antihypertensive therapy for patients with diabetes and proteinuria?	Accurate	Accurate				

R2 → R3 (claude)						
Question	Round 2	Round 3	Improved Accuracy	Consistency	No Improvement	Declined Accuracy
1- What is the impact of beta-blockers on glycemic control in diabetic patients with HTN?	Accurate	Accurate		●		
2-What are the differences in pharmacologic treatment recommendations for DM and HTN in elderly patients?	Accurate	Accurate		●		
3-What are the best exercise recommendations for patients with both DM and HTN?	Accurate	Accurate		●		
4-What are the first line medications for DM and HTN?	Accurate	Accurate		●		
5-What are the most common contraindications for DM and HTN medications?	Accurate	Accurate		●		
6- What is the recomended diet modification for DM and HTN?	Accurate	Accurate with missing information				●
7- What is the recomended diet modification for DM and HTN?	Accurate	Accurate		●		
8- What is the recomended diet modification for DM and HTN?	Inaccurate	Accurate	●			
9- What is the recomended diet modification for DM and HTN?	Accurate	Accurate with missing information				●

10- What is the recomended diet modification for DM and HTN?	Accurate	Accurate		●		
Q11What are the recommended frequencies for monitoring blood glucose, HbA1c, and blood pressure in patients with DM and/or HTN?	Accurate	Accurate with missing information				●
Q12 what are the medications recommended for hypertension and DM in pregnant persons?	Accurate	Accurate		●		
Q13 What are the guidelines address the management of DM and HTN medications in the context of chronic kidney disease ?	Accurate	Accurate		●		
Q14 What is the main treatment of what is the main treatment of DKA?	Accurate	Inaccurate				●
Q15 What is the main non-pharmacotherapy to treat HTN?	Accurate	Inaccurate				●
16.Which antihypertensive classes are preferred in patients with diabetes and evidence of nephropathy?	Accurate	Accurate		●		
17.What is the first choice treatment in patients with DM2 ?	Accurate	Accurate		●		
18.what is the treatment of choice in Gestational diabetes?<br>What is the best diet for patients with uncontrolled type 2 diabetes mellitus (DM2)?	Accurate	Accurate		●		
				●		

19.What is the preferred antihypertensive therapy for patients with diabetes and proteinuria?	Accurate	Accurate				
Q20. What is the preferred antihypertensive therapy for patients with diabetes and proteinuria?	Accurate	Accurate				

R2 → R3 (perplexity)						
Question	Round 2	Round 3	Improved Accuracy	Consistency	No Improvement	Declined Accuracy
1- What is the impact of beta-blockers on glycemic control in diabetic patients with HTN?	Accurate	Accurate		●		
2-What are the differences in pharmacologic treatment recommendations for DM and HTN in elderly patients?	Inaccurate	Accurate with missing information	●			
3-What are the best exercise recommendations for patients with both DM and HTN?	Accurate	Accurate		●		
4-What are the first line medications for DM and HTN?	Accurate	Accurate		●		
5-What are the most common contraindications for DM and HTN medications?	Accurate with missing information	Accurate with missing information			●	
6- What is the recomended diet modification for DM and HTN?	Accurate with missing information	Accurate with missing information			●	
7- What is the recomended diet modification for DM and HTN?	Inaccurate	Accurate	●			
8- What is the recomended diet modification for DM and HTN?	Inaccurate	Accurate with missing information	●			
9- What is the recomended diet modification for DM and HTN?	Accurate	Accurate		●		
10- What is the recomended diet modification for DM and HTN?	Accurate with missing information	Accurate with missing information			●	

Q11What are the recommended frequencies for monitoring blood glucose, HbA1c, and blood pressure in patients with DM and/or HTN?	Accurate with missing information	Accurate with missing information			●	
Q12 what are the medications recommended for hypertension and DM in pregnant persons?	Accurate	Accurate		●		
Q13 What are the guidelines address the management of DM and HTN medications in the context of chronic kidney disease ?	Accurate	Accurate		●		
Q14 What is the main treatment of what is the main treatment of DKA?	Inaccurate	Inaccurate			●	
Q15 What is the main non-pharmacotherapy to treat HTN?	Accurate	Accurate		●		
16.Which antihypertensive classes are preferred in patients with diabetes and evidence of nephropathy?	Accurate	Accurate		●		
17.What is the first choice treatment in patients with DM2 ?	Accurate	Accurate		●		
18.what is the treatment of choice in Gestational diabetes? What is the best diet for patients with uncontrolled type 2 diabetes mellitus (DM2)?	Accurate	Accurate		●		
19.What is the preferred antihypertensive therapy for patients with diabetes and proteinuria?	Accurate	Accurate		●		

Q20. What is the preferred antihypertensive therapy for patients with diabetes and proteinuria?	Accurate	Accurate				

R2 → R3 (consensus)

Question	Round 2	Round 3	Improved Accuracy	Consistency	No Improvement	Declined Accuracy
1- What is the impact of beta-blockers on glycemic control in diabetic patients with HTN?	Accurate with missing information	Accurate with missing information			●	
2-What are the differences in pharmacologic treatment recommendations for DM and HTN in elderly patients?	Accurate with missing information	Inaccurate				●
3-What are the best exercise recommendations for patients with both DM and HTN?	Accurate	Accurate		●		
4-What are the first line medications for DM and HTN?	Inaccurate	Inaccurate			●	
5-What are the most common contraindications for DM and HTN medications?	Inaccurate	Inaccurate			●	
6- What is the recomended diet modification for DM and HTN?	Accurate with missing information	Accurate with missing information			●	
7- What is the recomended diet modification for DM and HTN?	Accurate with missing information	Accurate	●			
8- What is the recomended diet modification for DM and HTN?	Accurate with missing information	Inaccurate				●
9- What is the recomended diet modification for DM and HTN?	Accurate with missing information	Accurate	●			

10- What is the recomended diet modification for DM and HTN?	Inaccurate	Accurate with missing information	●			
Q11What are the recommended frequencies for monitoring blood glucose, HbA1c, and blood pressure in patients with DM and/or HTN?	Accurate	Accurate with missing information				●
Q12 what are the medications recommended for hypertension and DM in pregnant persons?	Accurate with missing information	Inaccurate				●
Q13 What are the guidelines address the management of DM and HTN medications in the context of chronic kidney disease ?	Accurate	Inaccurate				●
Q14 What is the main treatment of what is the main treatment of DKA?	Accurate with missing information	Inaccurate				●
Q15 What is the main non-pharmacotherapy to treat HTN?	Accurate with missing information	Inaccurate				●
16.Which antihypertensive classes are preferred in patients with diabetes and evidence of nephropathy?	Accurate	Accurate		●		
17.What is the first choice treatment in patients with DM2 ?	Accurate	Accurate		●		
18.what is the treatment of choice in Gestational diabetes? What is the best diet for patients with uncontrolled type 2 diabetes mellitus (DM2)?	Accurate	Accurate		●		
19.What is the preferred antihypertensive therapy for	Accurate	Accurate		●		

patients with diabetes and proteinuria?						
20. What is the preferred antihypertensive therapy for patients with diabetes and proteinuria?	Accurate	Accurate				
